# Postprandial Effect of a High-Fat Meal on Endotoxemia in Arab Women with and without Insulin-Resistance-Related Diseases

**DOI:** 10.3390/nu7085290

**Published:** 2015-08-04

**Authors:** Dara A. Al-Disi, Nasser M. Al-Daghri, Nasiruddin Khan, Assim A. Alfadda, Reem M. Sallam, Mohammed Alsaif, Shaun Sabico, Gyanendra Tripathi, Philip G. McTernan

**Affiliations:** 1 Division of Biomedical Sciences, Warwick Medical School, University of Warwick, Coventry CV2 2DX, UK; E-Mails: dara.aldesi@warwick.ac.uk (D.A.A.-D.); s.l.sabico@warwick.ac.uk (S.S.); g.tripathi@warwick.ac.uk (G.T.); 2 Department of Community Health Sciences, College of Applied Medical Sciences, King Saud University, Riyadh 11451, Saudi Arabia; E-Mail: malsaif@ksu.edu.sa; 3 Biomarkers Research Program, Riyadh Biochemistry Department, College of Science, King Saud University, Riyadh 11451, Saudi Arabia; E-Mail: nasiruddin2006@gmail.com; 4 Prince Mutaib Chair for Biomarkers of Osteoporosis, Biochemistry Department, College of Science, King Saud University, Riyadh 11451, Saudi Arabia; 5 Obesity Research Center, College of Medicine, King Saud University, Riyadh 11461, Saudi Arabia; E-Mails: dralfadda@hotmail.com (A.A.A.); sallam_reem@yahoo.com (R.M.S.); 6 Department of Medicine, College of Medicine, King Saud University, Riyadh 11461, Saudi Arabia; 7 Clinical Chemistry Unit, Department of Pathology, College of Medicine, King Saud University, Riyadh 11461, Saudi Arabia

**Keywords:** endotoxin, type 2 diabetes mellitus, Arab women, high fat meal

## Abstract

This study determined the effects of a high-fat meal on circulating endotoxin and cardiometabolic indices in adult Arab women. The cohort consisted of 92 consenting Saudi women (18 non-diabetic (ND)) control subjects; Age 24.4 ± 7.9 year; body mass index (BMI) 22.2 ± 2.2 Kg/m^2^), 24 overweight/obese (referred to as overweight-plus (overweight^+^)) subjects (Age 32.0 ± 7.8 year; BMI 28.5 ± 1.5 Kg/m^2^) and 50 type 2 diabetes mellitus (T2DM) patients (Age 41.5 ± 6.2 year; BMI 35.2 ± 7.7 Kg/m^2^). All were given a high-fat meal (standardized meal: 75 g fat, 5 g carbohydrate, 6 g protein) after an overnight fast of 12–14 h. Anthropometrics were obtained and fasting blood glucose, lipids, and endotoxin were serially measured for four consecutive postprandial hours. Endotoxin levels were significantly elevated prior to a high-fat meal in the overweight^+^ and T2DM than the controls (*p* < 0.05). Furthermore, the postprandial cardiometabolic changes led to a more detrimental risk profile in T2DM subjects than other groups, with serial changes most notable in glucose, triglycerides, high density lipoprotein-cholesterol (HDL-cholesterol), and insulin levels (*p*-values < 0.05). The same single meal given to subjects with different metabolic states had varying impacts on cardiometabolic health. Endotoxemia is exacerbated by a high-fat meal in Arab subjects with T2DM, accompanied by a parallel increase in cardiometabolic risk profile, suggesting disparity in disease pathogenesis of those with or without T2DM through the altered cardiometabolic risk profile rather than variance in metabolic endotoxinaemia with a high-fat meal.

## 1. Introduction

The nutritional transition and the rapid urbanization in the Middle East has introduced energy-dense refined carbohydrates and increased saturated fat intake [1]. This transition has paralleled the increase in lifestyle-related chronic diseases such as obesity and type 2 diabetes mellitus (T2DM) [2,3].

Whilst obesity represents the single most influential risk factor for T2DM, weight gain itself is a result of a complex interaction between genetic, epigenetic, and environmental factors. Amongst the latter, a carbohydrate-rich high-fat diet can quickly drive the increased obesity mediated T2DM [4]. The major metabolic consequence of a high-fat diet is the negative effect on insulin action, where the regulatory mechanisms of body weight become impaired through lipotoxic effects as well as the increased low grade chronic systemic inflammatory response [5,6].

Previous models of diet-induced and genetic obesity has shown that adipose tissue presents an important source of pro-inflammatory adipocytokines, such as tumor necrosis factor α (TNF-α), interleukin-1 (IL-1), and interleukin-6 (IL-6) during weight gain [7,8]. These adipocytokines can have a simultaneous dual impact leading to insulin resistance through activation of the pro-inflammatory mechanisms as well as a direct impact on insulin signaling capacity [9]. As such, the development of the insulin resistance through a sustained energy dense diet promotes hyperinsulinaemic conditions, coupled with increased adipose tissue and ectopic fat accumulation [10].

The impact of a high-fat diet at the molecular level remains to be fully understood. However, postprandial lipidaemia has emerged as a potential candidate to promote an inflammatory response. Studies have shown that the ingestion of a single high-fat meal can mediate systemic increases of a wide range of inflammatory factors with noted activation of nuclear factor-κB (NF-κB) in leukocytes [11,12,13,14], a key transcription factor in the inflammatory cascade that regulates the transcription of numerous pro-inflammatory cytokines and adipocytokines [15,16]. To date, the cause of these postprandial inflammatory events remains poorly understood. One potential candidate factor is bacterial endotoxin (lipopolysaccharide (LPS)), a potent inflammatory bacterial antigen that is present in large quantities in the human gut [17]. Endotoxin circulates in the blood of healthy human subjects at low concentrations (between 1 and 200 pg/mL) [18]. However, clinical studies have implicated gut-derived endotoxin as a “primary insult” that activates the inflammatory state, contributing to metabolic disease, with cross sectional data showing elevated systemic endotoxin levels in obesity, T2DM, coronary artery disease and fatty liver disease with the ability to be influenced by changes in diet [19,20,21,22,23].

The aim of this study was therefore to determine the influence of a high-fat meal on changes in circulating endotoxin and whether this is altered in different metabolic disease states amongst Arab adult women.

## 2. Experimental Section

The study comprised of three groups of subjects: non-T2DM, lean subjects (control; Body Mass Index (BMI) 22.2 ± 2.2 Kg/m^2^; *n* = 18), overweight/obese (referred to as overweight-plus (overweight^+^)) subjects (overweight^+^ 28.5 ± 1.5 Kg/m^2^) subjects (*n* = 24), and patients with early onset of T2DM (BMI: 35.2 ± 7.7 Kg/m^2^; *n* = 50). All subjects were Saudi pre-menopausal women, randomly selected from different primary care centers (PCCs) of Riyadh, Saudi Arabia, nonsmokers, with a normal resting electrocardiogram (ECG) and blood pressure, and with no history of vascular disease. In addition, subjects with known long-standing diabetes and/or receiving anti-diabetic medication, those with fasting glucose levels > 11 mmol/L, or with fasting triglycerides levels > 4 mmol/L were excluded from the study. Ethical approval was granted by the Ethics Committee of King Saud University (No. 10-173), Riyadh, Kingdom of Saudi Arabia, prior to the commencement of the research and all patients gave written consent.

Screening fasting blood tests at baseline were performed to qualify subjects for the study and to assess glucose control as well as lipid profiles. In addition, all subjects had their weight, height, waist and hip circumferences measured. Weight (in kilograms) was measured in light clothing to the nearest 0.1 kg. Height was measured using a digital stadiometer to the nearest centimeter. Waist circumference was measured at the level of the iliac crest at the end of normal respiration, and hip was measured at the widest circumference around the buttocks using measuring tape. BMI, as well as waist-to-hip ratios (WHR), were calculated. Blood samples were taken from the right or left antecubital vein in the sitting position. Blood pressure was checked with a blood pressure monitor on the left arm using a standard protocol. All subjects (*n* = 92) with and without T2DM were given a high-fat meal (standardized meal: 75 g fat, 5 g carbohydrate, 6 g protein per m^2^ body surface area corresponding to 700 Kcal/m^2^ [24]) after an overnight fast of 12–14 h.

### 2.1. In Vivo Assessment of the Biochemical Profile

Blood samples were drawn via cannula at baseline (0 h) and postprandially (1, 2, 3, and 4 h), and endotoxin and lipid levels were measured. Serum glucose and lipid profile were measured routinely using a glucose oxidase method in an autoanalyzer (Konelab, Espoo, Finland). Serum-free insulin concentrations were determined by electro-chemiluminescence method (COBAS-E-411; Roche Diagnostics, Mannheim, Germany). Homeostasis model assessment for insulin resistance (HOMA-IR) was then calculated for all patients using the HOMA formula:

HOMA-IR = fasting insulin (mU/L) × fasting plasma glucose (mmol/L)/22.5 [25].
(1)

### 2.2. Analysis of Circulating Endotoxin

Serum endotoxin was analyzed using a commercially available QCL-1000 LAL End Point Assay (Lonza, Allendale, NJ, USA). The assay, and the values given by the manufacturer for intra-assay coefficient of variation (CV) (3.9% ± 0.46%) and inter-assay CV (9.6% ± 0.75%), have been validated as detailed previously by this research group [16].

### 2.3. Statistical Analysis

Data were analyzed using SPSS version 16.5 (SPSS, Chicago, IL, USA). All continuous variables were presented as mean ± standard deviation and were normalized prior to parametric analyses. For comparison between groups, Analysis of Variance (ANOVA) with Tukey *post-hoc* analysis and Kruskal-Wallis (for triglycerides) were used. For comparison overtime, repeated measures ANOVA and Friedman’s two-way analysis of variance (for triglycerides, insulin, HOMA-IR and endotoxin) were used. For associations between endotoxin and variables of interest postprandial, Spearman bivariate correlations were utilized. Significance was set at *p* < 0.05.

## 3. Results

The clinical and metabolic characteristics were determined (Table 1) according to groups. Subjects with T2DM had a mean duration of diabetes of 2.04 years. Furthermore, it noted that there were significant differences in age and BMI across the cohort (Table 1). As expected, the T2DM group also had the highest anthropometric indices (BMI, waist and hip circumferences as well as WHR) than the overweight^+^ group and controls, with the overweight^+^ group being significantly higher than the controls in all indices as well. As anticipated, subjects with T2DM had significantly higher serum glucose levels (7.9 ± 2.73 mmol/L) than the overweight^+^ (4.7 ± 0.41 mmol/L, *p* < 0.01) and control subjects (4.81 ± 0.86 mmol/L, *p* < 0.001). The subjects with T2DM also had significantly higher serum triglycerides (1.9 ± 1.0 mmol/L), total cholesterol (5.4 ± 1.07 mmol/L) and low density lipoprotein-cholesterol (LDL-cholesterol) (3.66 ± 0.8 mmol/L), as well as the lowest in mean high density lipoprotein-cholesterol (HDL-cholesterol) (0.96 ± 0.21 mmol/L) (all *p* < 0.001). Serum glucose and lipid levels of the overweight^+^ and control groups were not significantly different from one another (Table 1).

**Table 1:** Clinical and metabolic characteristics of subjects according to group.

	Control	Overweight^+^	T2DM	*p*-Value
*N*	18	24	50	
Age (years)	24.4 ± 7.9	32.0 ± 7.8 *	41.5 ± 6.2 *^!^	<0.001
T2DM Duration (years)	--	--	2.04 (0–9)	
BMI (Kg/m^2^)	22.2 ± 2.2	28.5 ± 1.5 *	35.2 ± 7.7 *^!^	<0.001
Waist (cm)	80.6 ± 7.2	95.8 ± 7.4 *	112.3 ± 13.4 *^!^	<0.001
Hip (cm)	98.7 ± 7.3	109.7 ± 5.0 *	117.1 ± 11.6 *^!^	<0.001
WHR	0.8 ± 0.05	0.9 ± 0.05 *	1.0 ± 0.07 *^!^	<0.001
Glucose (mmol/L)	4.8 ± 0.9	4.7 ± 0.4	7.9 ± 2.7 *^!^	<0.001
LDL-Cholesterol (mmol/L)	2.8 ± 0.6	2.8 ± 0.7	3.7 ± 0.8 *^!^	<0.001
Triglycerides (mmol/L) #	1.0 ± 0.4	1.3 ± 0.8	1.9 ± 1.0 *^!^	0.001
Total Cholesterol (mmol/L)	4.2 ± 0.7	4.5 ± 01.0	5.4 ± 1.1 *^!^	0.003
HDL-Cholesterol (mmol/L)	1.3 ± 0.2	1.1 ± 0.4	0.96 ± 0.2 *^!^	<0.001

Data presented as mean ± standard error; # denotes non-Gaussian distribution; *p*-values at extreme right denotes over-all significance according to group; “*” denotes significance as compared with control subjects; “^!^” denotes significance as compared with overweight^+^ group; “--” denotes absence of T2DM in subjects; *p*-value significant at < 0.05. Analysis of Variance (ANOVA) with Tukey *post-hoc* analysis and Kruskal-Wallis (for triglycerides) tests were used (T2DM: Type 2 diabetes Mellitus; BMI: Body mass index; WHR: Waist hip ratio; LDL: Low density lipoprotein; HDL: High density lipoprotein).

### 3.1. Effects of High-Fat Meal in Different Groups

Blood samples were taken over time following a high-fat meal to assess changes in metabolic indices and endotoxin changes in the three cohorts. In the T2DM group, mean glucose level was highest at 0 h and lowest after 4 h, which was significantly lower than other hours with a noted stepwise reduction over time (Table 2). In both the control and overweight^+^ groups, no significant changes in glucose were noted over time (Table 2). In contrast to glucose levels, the triglyceride levels for all groups followed an increasing trend over time. The triglyceride levels in the subjects with T2DM was highest postprandially between 3 and 4 h, and was significantly higher than hours 0–2 (*p* < 0.01). In the overweight^+^ group, triglyceride levels were highest at 3 h than 4 h, with a stepwise increase from baseline over time until 3 h. Comparing all groups, the subjects with T2DM had significantly higher mean triglyceride levels as compared with either the overweight^+^ or control groups (*p* < 0.01). No significant changes were noted with total cholesterol over time.

In the subjects with T2DM, HDL-cholesterol was lowest after 4 h and was significantly lower than hours 0–3 (*p* < 0.01) with, again, a stepwise reduction in HDL-cholesterol level (Table 2). Similarly, the HDL-cholesterol levels in the overweight^+^ group were lowest at hour 4 than the previous hours 0–2 (*p* < 0.01). In contrast, the HDL-cholesterol levels of the control group were significantly higher than both the T2DM and overweight^+^ groups (*p* < 0.01).

In the T2DM group, LDL-cholesterol levels at baseline were significantly higher than hours 1–4 (*p* < 0.05). In the overweight^+^ group, LDL-cholesterol levels at baseline were significantly higher than hours 2–4 (*p* < 0.05). In the control group, LDL-cholesterol levels at baseline was significantly lower than hours 1 and 2 (*p* < 0.05) returning to baseline levels after that. T2DM had significantly higher LDL-cholesterol levels than either the control or overweight^+^ group.

**Table 2:** Metabolic changes pre- and post-high-fat meal.

	0 h	1 h	2 h	3 h	4 h
**GLUCOSE (mmol/L)**					
T2DM (*N* = 50)	7.9 ± 2.7	7.8 ± 2.5	7.5 ± 2.5 *	7.29 ± 2.7 *^!§^	7.0 ± 2.8 *^!§†^
Overweight^+^ (*N* = 24)	4.7 ± 0.4	4.6 ± 0.4	4.6 ± 0.4	4.6 ± 0.39	4.63 ± 0.7
Control (*N* = 18)	4.8 ± 0.86	5.1 ± 2.3	5.02 ± 1.7	4.76 ± 1.51	4.79 ± 1.6
**TRIGLYCERIDES (mmol/L)** #					
T2DM (*N* = 50)	1.9 ± 1.0	1.8 ± 0.7	2.4 ± 0.9 *^!^	2.7 ± 1.1 *^!§^	2.7 ± 1.3 *^!§^
Overweight^+^ (*N* = 24)	1.3 ± 0.8	1.4 ± 0.8	1.7 ± 0.9 *^!^	2.0 ± 1.1 *^!§^	1.9 ± 1.3 *^!§^
Control (*N* = 18)	1.0 ± 0.4	1.2 ± 0.6	1.4 ± 0.9	1.44 ± 0.91	1.54 ± 1.0
**TOTAL CHOLESTEROL** (mmol/L)					
T2DM (*N* = 50)	5.4 ± 1.1	5.3 ± 1.0	5.4 ± 1.1	5.3 ± 1.1	5.4 ± 1.1
Overweight^+^ (*N* = 24)	4.5 ± 1.0	4.5 ± 0.9	4.4 ± 0.8	4.4 ± 0.8	4.4 ± 1.0
Control (*N* = 18)	4.2 ± 0.7	4.1 ± 0.7	4.1 ± 0.6	4.1 ± 0.6	4.2 ± 0.7
**HDL-CHOLESTEROL** (mmol/L)					
T2DM (*N* = 50)	1.0 ± 0.2	1.0 ± 0.2	0.9 ± 0.2	0.9 ± 0.2 *^!^	0.89 ± 0.2 *^!§†^
Overweight^+^ (*N* = 24)	1.2 ± 0.3	1.1 ± 0.3	1.1 ± 0.3	1.1 ± 0.4 *^!^	1.1 ± 0.4 *^!§^
Control (*N* = 18)	1.3 ± 0.2	1.2 ± 0.3	1.2 ± 0.3	1.2 ± 0.3	1.2 ± 0.3
**LDL-CHOLESTEROL** (mmol/L)					
T2DM (*N* = 50)	3.7 ± 0.8	3.6 ± 0.8	3.4 ± 0.9 *	3.2 ± 0.9 *	3.3 ± 0.9 *
Overweight^+^ (*N* = 24)	2.8 ± 0.7	2.7 ± 0.6	2.5 ± 0.6 *	2.4 ± 0.6 *	2.4 ± 0.6 *
Control (*N* = 18)	2.7 ± 0.6	2.6 ± 0.6*	2.6 ± 0.6 *	2.6 ± 0.6	2.7 ± 0.6
**Endotoxin** (EU/mL)					
T2DM (*N* = 50)	3.4 ± 0.8	3.0 ± 0.8	3.4 ± 0.9 ^!^	3.5 ± 0.9 ^!^	3.6 ± 0.9 ^!^
Overweight^+^ (*N* = 24)	3.0 ± 0.5	2.9 ± 1.4	3.5 ± 0.9	3.8 ± 1.6	3.5 ± 1.9
Control (*N* = 18)	1.5 ± 0.1	1.8 ± 0.1 *	1.7 ± 0.8 *	1.9 ± 0.2 *	2.1 ± 0.2 *
**Insulin** (IU/mL) #					
T2DM (*N* = 50)	11.7 ± 5.5	21.9 ± 17.7 *	19.3 ± 12.6 *	16.2 ± 12.3 *	14.3 ± 8.2 *
Overweight^+^ (*N* = 24)	5.0 ± 3.4	15.6 ± 16.2 *	16.9 ± 16.0 *	13.1 ± 8.0 *	10.3 ± 5.3 *
Control (*N* = 18)	5.8 ± 0.64	10.3 ± 1.7	9.6 ± 3.4	8.2 ± 1.6	10.2 ± 2.6
**HOMA-IR** #					
T2DM (*N* = 50)	3.7 ± 2.0	8.7 ± 12.0 *^‡^	6.6 ± 6.0 *^‡^	5.6 ± 5.4 ^‡^	3.9 ± 2.4
Overweight^+^ (*N* = 24)	1.12 ± 0.7	3.5 ± 4.1 *	3.9 ± 4.1 *	2.8 ± 1.9 *	2.1 ± 1.2
Control (*N* = 18)	1.3 ± 0.2	1.9 ± 0.4	2.1 ± 0.7 *	1.9 ± 0.4	2.2 ± 0.6

# denotes Non-Gaussian distribution; * denotes significance compared with 0 hour; ^!^ denotes significance compared with 1; ^§^ denotes significance compared with 2; ^†^ denotes significance compared with 3; ^‡^ denotes significance compared with 4; *p* significant at ≤0.05. Repeated measures ANOVA and Friedman’s two-way analysis of variance (for triglycerides, insulin, HOMA-IR and endotoxin) were the tests used (T2DM: Type 2 diabetes Mellitus; Overweight^+^: overweight/obese subjects referred to as overweight-plus; LDL: Low density lipoprotein; HDL: High density lipoprotein; HOMA-IR: Homeostasis model assessment for insulin resistance; ANOVA: analysis of Variance).

The highest endotoxin levels were noted at hour 4 in all groups and was statistically significant compared with hour 1 in the T2DM group and baseline in the control group (*p* < 0.01). No significant changes were observed in the overweight^+^ group. Both the T2DM and overweight^+^ groups had significantly higher endotoxin levels than the control subjects. Insulin levels were also noted to change over time in the subjects with T2DM, with the highest peak insulin levels noted at hours 1–2, lowest at hours 3–4, and baseline fasted insulin levels being significantly lower as compared to insulin levels across time (*p* < 0.01). This similar pattern was observed in the overweight^+^ and control groups, with the baseline noting the lowest insulin levels over time. Between groups, the insulin level in the T2DM group was significantly higher than either the overweight^+^ or control group as expected (*p* < 0.01). Lastly, in both the T2DM and overweight^+^ groups, HOMA-IR was lowest at baseline at the fourth hour with the T2DM group showing the highest mean HOMA-IR post 1 h feed and the overweight^+^ group at hour 2. No significant changes in HOMA-IR were observed in the control group over time. Between groups, the HOMA-IR in subjects with T2DM was significantly higher than those of the overweight^+^ and control groups (*p* < 0.01).

### 3.2. Associations of Metabolic Parameters to Endotoxin after a High-Fat Meal

In all subjects, endotoxin was positively associated with LDL cholesterol (*R* = 0.38; *p* < 0.05) and this was observed 3 h after a high-fat meal. In the subjects with T2DM, endotoxin was significantly associated with triglycerides at 3 and 4 h postprandial (*R* values 0.52 and 0.50; *p* < 0.05, respectively). In the overweight^+^ subjects, endotoxin was highly associated with triglycerides (*R* = 0.63; *p* < 0.05) and total cholesterol (*R* = 0.71; *p* < 0.05) at baseline. Whilst in the control subjects no associations were observed at baseline or over time (Table 3).

**Table 3:** Bivariate associations between lipids, glucose and endotoxin.

ALL SUBJECTS																												
Glucose						Triglycerides						Total Cholesterol						HDL-Cholesterol						LDL-Cholesterol				
0	1	2	3	4		0	1	2	3	4		0	1	2	3	4		0	1	2	3	4		0	1	2	3	4
−0.17	0.04	0.13	0.08	0.00		−0.13	0.10	0.14	0.22	0.20		0.16	−0.08	0.00	−0.07	−0.21		−0.16	0.15	−0.01	−0.08	−0.06		0.22	−0.06	0.05	0.38	−0.12
**T2DM SUBJECTS (*N* = 50)**																												
−0.20	0.23	0.29	0.23	0.08		−0.12	0.32	0.26	0.52	0.50		0.18	0.04	0.02	0.0	−0.08		−0.28	-0.15	−0.14	−0.30	−0.05		0.30	0.02	0.005	−0.06	−0.19
**OVERWEIGHT^+^ SUBJECTS (*N* = 24)**																												
0.23	0.14	0.08	0.0	−0.18		0.63	0.04	0.08	0.26	0.25		0.71	−0.08	0.10	−0.13	−0.33		−0.13	0.41	0.13	−0.07	−0.18		0.36	0.14	0.43	0.64	0.28
**CONTROL SUBJECTS (*N* = 18)**																												
−0.36	0.15	0.22	0.24	0.14		−0.20	0.16	0.30	−0.14	−0.17		--	0.01	0.14	−0.05	−0.26		−0.28	−0.20	−0.39	−0.16	−0.31		−0.02	0.08	0.24	0.07	−0.09

Data presented as coefficient (*R*); bold and red denotes significance at *p* < 0.05. Spearman correlation tests were used (T2DM: Type 2 diabetes Mellitus; Overweight^+^: overweight/obese subjects referred to as overweight-plus; Control Subjects: Non-daibetic lean individiuals; LDL: Low density lipoprotein; HDL: High density lipoprotein). “--” denotes absence of T2DM in subjects.

## 4. Discussion

The present results affirm and extend our knowledge on the impact of a high-fat meal in different metabolic states. Specifically, circulating endotoxin levels were significantly raised during a high-fat meal in overweight^+^ and T2DM metabolic states, with the impact of cardiometabolic changes imposing a more detrimental risk profile in T2DM subjects than either the overweight^+^ or the non-diabetic lean control subjects as assessed by glucose, triglycerides, insulin and HOMA-IR and lower HDL postprandially. The results also highlighted that the same single meal given to subjects in different metabolic states had a different impact on cardiometabolic health. As such, daily repetition of this type of meal could have a much more damaging effect in the subjects with T2DM, closely followed by the overweight^+^ subjects; whilst lean and non-diabetic subjects appear to better handle the insult of a high-fat meal, metabolically.

Despite the impact of the meal it was also identified that prior to the high-fat meal, the presence of sub-chronic inflammation was already apparent, with circulating endotoxin being highest in subjects with T2DM, affirming prior studies that suggest that endotoxin levels are altered in the presence of insulin-resistant diseases [16,19,20,21,22,26]. Previous studies have also shown that a high-fat meal, regardless of the individual’s metabolic status, can induce inflammatory changes [27,28]. In this study, circulating endotoxin, a potential mediator of a low grade chronic inflammatory response, was comparably raised in both the overweight^+^ and T2DM states, unlike previous studies in South Asians [26], suggesting that Arabs in the overweight^+^ state may at least be at even higher metabolic risk, which could help to explain the country’s current higher metabolic disease per capita.

Elevated endotoxin levels amongst Middle-Eastern patients with T2DM are also consistent across ethnic groups, such as Africans [29], Chinese [30], Caucasians [16,31], and South Asians [22,23]. Whilst endotoxin is seen as an important mediator of sub-clinical inflammation, Piya and colleagues have suggested that, ultimately, the gut flora may act as an essential determinant of the sub-chronic inflammation induced by obesity and T2DM, and that endotoxin may act as a systemic insult that triggers the inflammatory cascade [32]. In Saudi women, a high-fat meal given to both overweight+ and T2DM groups increased endotoxin levels from a higher baseline in these groups without a clear difference between them, in contrast to previous studies in South Asians [26]. Animal studies have shown that continuous infusion of endotoxin increases gut permeability, as does high-fat dietary feeding, therefore, one possible explanation for the difference could be due to the dietary differences in fat consumption that the obese subjects eat in Saudi Arabia as compared in UK [33].

Animal studies involving *ob/ob* and *db/db* mice have demonstrated a leaky gut which is considered to be related to the impact insulin resistance on gut endothelium [33]. As such, our overweight^+^ and T2DM subjects may have a more frequent snack habit, which consequentially affects how they subsequently handled the high-fat meal, which could be different from the white Caucasians given such a diet. Furthermore, ethnicity may clearly impact on individual sub-clinical inflammatory risk. Previous studies have shown that endotoxin can be stratified by gender and ethnicity; therefore, these data could help explain why different ethnicities have a variable cardiometabolic risk due to post-feeding increases in endotoxin [21].

The current study also considered the impact of a high-fat meal on elevating glucose and cholesterol levels, both considered important in the development of coronary artery disease, coupled with obesity and T2DM. The changes observed postprandially in the lipid profile and insulin in this study, resulted in lipidaemia regardless of their metabolic status and this is consistent with previous studies [26,34,35,36]. Prior analysis of the effects of a high saturated fat meal in other studies indicates that lipidaemia can mediate deleterious changes at the proteome and genome levels producing a pro-coagulant state [37]. Furthermore, it appears from other studies that triglycerides show the most dynamic changes during the postprandial phase compared to other lipids, regardless of the individual’s metabolic status, although it appears dependent on the type of fat diet used [38]. Clinically, this is relevant. As observed in the present and other studies, higher postprandial hypertriglyceridemia and hyperlipidemia are observed amongst subjects with T2DM than their healthier counterparts [39]. Therefore, dietary strategies defining the type of dietary fat for patients, to lower saturated fat content, appears important to lead to better management of the insulin response and increased fat oxidation, both important determinants of cardiometabolic risk in overweight and T2DM patients [40].

The different cardiometabolic risk factor changes in pre- and post-feeding showed that the T2DM group had significantly higher glucose, triglycerides, insulin, and HOMA-IR, as well as significantly lower HDL-cholesterol throughout the high-fat challenge as compared with overweight^+^ and control subjects. Whilst these significant differences were expected, since the baseline levels of T2DM subjects were already higher than other groups, the persistence of dysmetabolism in the T2DM group suggests that already deranged baseline glycemic and lipid parameters could be exacerbated further following a fat intake [41,42]. The postprandial increments in glucose and lipids observed confirms previous studies with women and even adolescents with T2DM [43,44,45] and also reaffirms the requirement to effectively manage postprandial glucose and lipid response in T2DM subjects through diet intervention to lower cardiovascular disease risk [42,46,47].

The authors acknowledge the significant variation in the mean age of the groups and this may have limited the present findings, taking into account the evidence that postprandial lipemia may be associated with age [48]. Nevertheless, comparisons were done mostly within groups and not independent of one another whilst observing for postprandial similarities and differences in patterns according to groups. Furthermore, the small and unequal sample size per group may have affected the few significant associations elicited out of a hundred possible correlations conducted, suggesting that these associations need to be further validated using a larger cohort per group.

## 5. Conclusions

In conclusion, our findings highlight that subjects with increased adiposity and or T2DM are at increased cardiometabolic risk given a high-fat meal, but particularly so in the subjects with T2DM. Furthermore, irrespective of their diabetic status, endotoxin levels, postprandial, were higher than control subjects. As such, the findings suggest that the disparity in lipidaemia, as opposed to endotoxin, post-meal, may exacerbate the pathogenesis of cardiovascular disease in Arab women in the long-term on this type of diet. Therefore, dietary interventions that can reduce the glycaemic and lipidomic response, as well as improving the gut microbiota for better gut barrier function, appears important in Saudi patients who have increased weight gain or a T2DM status.
